# Metabolomics analysis of herb-partitioned moxibustion treatment on rats with diarrhea-predominant irritable bowel syndrome

**DOI:** 10.1186/s13020-019-0240-2

**Published:** 2019-05-08

**Authors:** Xianwei Lin, Xia Liu, Jingjing Xu, Kian-Kai Cheng, Jianan Cao, Tao Liu, Qiong Liu, Huan Zhong, Guiping Shen, Jiyang Dong, Xiaorong Chang

**Affiliations:** 1 Department of Electronic Science, Fujian Provincial Key Laboratory for Plasma and Magnetic Resonance, Xiamen University, Xiamen, 361005 China; 2 College of Acupuncture and Moxibustion, Hunan University of Chinese Medicine, Changsha, 410208 China; 3 Innovation Centre in Agritechnology, Universiti Teknologi Malaysia, 84600 Muar, Johor Malaysia

**Keywords:** Irritable bowel syndrome, Herb-partitioned moxibustion, Metabolomics, Nuclear magnetic resonance

## Abstract

**Background:**

Irritable bowel syndrome (IBS) is a common functional gastrointestinal disorder, which is commonly treated with antidiarrhoeal, antispasmodics, serotonergic agents or laxative agents. These treatments provide relief for IBS symptoms but may also lead to undesired side effects. Previously, herb-partitioned moxibustion (HPM) treatment has been demonstrated to be effective in ameliorating symptoms of IBS. However, the underlying mechanism of this beneficial treatment is yet to be established. The aim of the current study was to systematically assess the metabolic alterations in response to diarrhea-predominant IBS (IBS-D) and therapeutic effect of HPM.

**Methods:**

Proton nuclear magnetic resonance spectroscopy (^1^H NMR)-based metabolomics approach was used to investigate fecal and serum metabolome of rat model of IBS-D with and without HPM treatment.

**Results:**

The current results showed that IBS-induced metabolic alterations in fecal and serum sample include higher level of threonine and UDP-glucose together with lower levels of aspartate, ornithine, leucine, isoleucine, proline, 2-hydroxy butyrate, valine, lactate, ethanol, arginine, 2-oxoisovalerate and bile acids. These altered metabolites potentially involve in impaired gut secretory immune system and intestinal inflammation, malabsorption of nutrients, and disordered metabolism of bile acids. Notably, the HPM treatment was found able to normalize the Bristol stool forms scale scores, fecal water content, plasma endotoxin level, and a number of IBS-induced metabolic changes.

**Conclusions:**

These findings may provide useful insight into the molecular basis of IBS and mechanism of the HPM intervention.

**Electronic supplementary material:**

The online version of this article (10.1186/s13020-019-0240-2) contains supplementary material, which is available to authorized users.

## Background

Irritable bowel syndrome (IBS) is a chronic functional gastrointestinal disorder [1] characterized by stomachache, abdominal distension and altered bowl evacuation habit, along with abnormal fecal character [2]. The pathophysiology of IBS is multifactorial including gut motility, visceral hypersensitivity, mucosal micro-inflammation, increases in intestinal permeability, psychosocial factors, and brain-gut dysfunctions [3]. Based on predominant bowel habit and Rome III criteria [4], IBS can be classified into four subgroups including diarrhea-predominant IBS (IBS-D), constipation-predominant IBS (IBS-C), mixed diarrhea and constipation IBS (IBS-M) and unspecific IBS (IBS-U). Although IBS has a very low mortality rate, it poses a considerable medical, social and economic burden on individuals and societies [5].

The conventional medications for IBS provide relief for symptoms, however it may also lead to unwanted side effects. For example, a number of antidiarrhoeal and antispasmodics agents may lead to constipation; some serotonergic agents (e.g. 5-hydroxytryptamine_3_ and 5-hydroxytryptamine_4_) may result in severe constipation and ischaemic colitis; some laxative agents may induce increased bowel gas and abdominal distention [6]. Traditional Chinese medicine (TCM) also offers complementary and alternative therapies for IBS, and they were found to improve the IBS symptom with good feedback from patients [7]. Among the TCM treatments, herb-partitioned moxibustion (HPM) is a characteristic external therapy that has been repeatedly found to confer curative effect in ameliorating IBS symptoms [8]. However, the therapeutic mechanism of HPM on IBS is still unknown.

As a systematic approach, metabolomics has been used to study gastrointestinal disorders including IBS and metabolic response following interventions [9]. The ^1^H high-resolution NMR spectroscopy has been widely used in metabolomics due to its nondestructive characteristics, high reproducibility and the requirement of minimal sample preparation [10]. In NMR-based metabolomics, peaks from a metabolite with given structural formula are determined by chemical shift (ppm) and intensities (proportional to the number of hydrogen atoms) in a ^1^H-NMR spectrum without the requirement of chromatographic separation and sample derivatization as in mass spectrometry-based metabolomics. The holistic strategy of NMR-based metabolomics is consistent with the systematic thinking of TCM, and thus may serve as a potential scientific platform to uncover the mechanism of evidence-based Chinese medicine including acupuncture and moxibustion [11–13]. In the present study, the blood serum and feces specimen obtained from a rat model of IBS-D and the metabolic impact of HPM treatment were investigated by a combination of high resolution NMR spectroscopy and multivariate statistical analysis. The aim of this study was to investigate the metabolic basis of HPM treatment in improving symptoms of IBS.

## Methods

The Minimum Standards of Reporting Checklist (Additional file 1) contains details of the experimental design, and statistics, and resources used in this study.

### Ethical statement

Animal care and experimental procedures used in the current study were approved by the Institutional Animal Care and Use Committee of Hunan University of Chinese Medicine (Ethics No. SCXK 2011-0003). This study was conducted in accordance with local guidelines provided by National Institutes of Health for the Care and Use of Laboratory Animals and all efforts were made to minimize suffering of animals.

### Animal handling

In the present study, forty male Sprague–Dawley rats (aged 4 weeks with body weight 180 ± 10 g) were housed individually in metabolism cages and provided with food and water ad libitum. The animal room was under controlled condition (temperature 22 ± 1 °C, humidity 40–60% and a 12-h light–dark cycle). After acclimatization for 1 week, all rats were divided randomly into normal control rats (NC, *n* = 10) and other rats (*n *= 32) which were used for IBS modelling.

After being fasted for 24 h, all rats were deeply anaesthetized with a ether (300 mg/kg). The normal rats were injected with 0.9% saline solution (5 mg/kg) into the colon via PE90 tubing inserted to a depth of 8 cm from the anus. The IBS rat models were induced by colorectal administration of 4% acetic acid (5 mg/kg) in a similar manner to the normal rats followed by washing with 0.01 M phosphate buffer saline (5 mg/kg) [14]. Seven days later, the IBS rats were treated with restraining stress for 2 weeks (1 h per day). These procedures are considered a reliable method for induction of IBS-D in rats [15]. Then, the rats with confirmed IBS-D were randomly divided into three groups, including untreated IBS rats (IBS group, *n *=11), or IBS rats that received herb-partitioned moxibustion at traditional acupoints (HPM group, *n *= 10) or received pinaverium bromide treatment (PBT group, *n *=11) [16].

### Herb-partitioned moxibustion

The herbal powder formula used in the HPM treatment was as follow: *Baizhu* (*Rhizoma atractylodis*) (500 g), *Baishao* (*Radix paeoniaealba*) (500 g), *Chenpi* (*Pericarpium citrireticulatae*) (500 g), and *Fangfeng* (*Radix saposhnikoviae divaricatae*) (500 g). The combination of these four herbs is used in the “Tongxieyaofang”, a traditional Chinese medical prescription widely adopted in the clinical treatment of IBS-D [17]. Based on TCM understanding, the herbs collectively help to regulate liver function and rectify the spleen functions.

These traditional Chinese medicines were mixed, ground into a fine powder and stored in dry condition at room temperature (25 °C). Then, 2.5 g of powder was mixed with 3 g of vinegar into herbal paste, and then shaped into herbal cake (1 cm in diameter, 0.3 cm in height) using a herbal cake mold. Moxi cones (10 mg in weight, 0.5 cm in diameter, 1.5 cm in height) were hand-made by using refined mugwort floss (Huatuo, Suzhou, China).

Rats of the HPM group received the same fixation and hair located around the acupoints was cut off to expose the local skin before treatment. The acupoints of bilateral *Ganyu* (BL18), *Piyu* (BL20), *Zusanli* (ST36), *Zhangmen* (LR13) and *Qimen* (LA14) were selected for stimulation as these acupoints were found effective in relieving IBS symptoms in clinical practice [18]. According to the “Experimental Acupuncture and Moxibustion Science” combined with anthropomorphic analogy, *Ganshu* (BL18) acupoint is located below the ninth thoracic vertebra and 5 mm lateral to posterior midline. *Pishu* (BL20) is below the twelfth thoracic vertebra and 5 mm lateral to posterior midline. *Zusanli* (ST36) is located 5 mm below the fibular head and lateral to the anterior tubercle of the tibia. *Zhangmen* (LR13) is in front of the eleventh rib. *Qimen* (LA14) is located in the sixth intercostals space. The moxa cones were fixed to the herbal cake that was placed directly onto the acupoints. Five acupoints were stimulated (a 20-min session per day) one by one for 2 weeks. Burning injury was carefully avoided in the process by whisking way the burning ash in a timely manner.

For the PBT group, the solution of pinaverium bromide, 50 mg/tablet (Abbott, USA), was prepared at the concentration of 13.5 mg/kg and was fed in dose of 10 mL/kg by lavage once per day.

### Fecal water content and plasma endotoxin

After the treatment, stool forms of the rats were observed and scored according to the Bristol Stool Form (BSF) scale [19]. Fecal water content (FWC) was also examined to estimate colonic motility as a validated index. Briefly, the stool was weighed after collection, and weighed again after drying in the oven. FWC was calculated as follow: water content (%) = 100 × (wet weight − dry weight)/wet weight. Besides, plasma samples were collected for determination of endotoxin by using a commercially available kit for tachypleus amebocyte lysate (TAL) technique (Zhanjiang A&C Biological Ltd, Zhanjiang, China).

### Biological sample preparation

After the 2-week treatment course, fecal samples from all rats were collected, snap-frozen in liquid nitrogen and stored at − 80 °C until further metabolite extraction. The aqueous metabolites in fecal samples were extracted according to the protocol reported by Shao [20]. Thawed stool sample weighed 200 mg was homogenized with 1.50 mL phosphate buffer solution (1.0 M K_2_HPO_4_/NaH_2_PO_4_, pH 7.4, 99.9% D_2_O) containing 0.3 mM TSP (3-(trimethylsilyl) propionic-2,2,3,3-d4 acid sodium salt) as an internal reference standard and vortexed for 60 s. The mixed slurry was subjected to freeze–thaw (3 times) cycle by liquid nitrogen and followed by ultrasonication with a cycle of 20 s—vortexing − 10 s-waiting (20 times) in an ice bath. After 15 min of centrifugation (10,000×*g*, 4 °C), 600 μL of the supernatant was transferred to centrifugal tube and remaining residue was further subjected to the above-mentioned procedure again. An equal volume of the second extracts from the same samples were mixed with the first extracts and then centrifuged at 10,000×*g* for 15 min at 4 °C. A total of 550 μL supernatant was transferred into 5 mm nuclear magnetic resonance (NMR) tube (Norell, ST50-7, USA) for NMR experiment. In the current study, we focused on the changes in body metabolism induced by moxibustion on the IBS-D model. The lipophilic metabolites in feces were not included in our analysis as they are mainly come from exogenous lipids intake of food and gut microbiome.

For blood samples, whole blood was obtained from abdominal aorta of the animals using a catheter and left to clot at room temperature (22 °C) for 1 h. For serum sample, a volume of 400 μL was mixed with 200 μL phosphate buffer solution (90 mM K_2_HPO_4_/NaH_2_PO_4_, pH 7.4, 99.9% D_2_O) without TSP. Then the mixture was transferred to centrifugal tube and centrifuged at 10,000×*g* for 15 min at 4 °C. A total of 550 μL supernatant was transferred into 5 mm NMR tube for NMR experiment.

### NMR experiments

^1^H NMR spectra of the fecal and serum samples were acquired at 296 K using a Bruker 600 MHz NMR spectrometer (Bruker Biospin, Germany) equipped with a cryogenic inverse detection probe. The NMR spectra of fecal samples were recorded using the NOESYPR1D pulse sequence [21]. For the serum sample, an additional Carr–Purcell–Meiboom–Gill (CPMG) spin-echo pulse train [22] was incorporated into the NOESYPR sequence with a relaxation time (2 nτ) of 100 ms and an echo time (τ) of 250 μs. A total of 64 free induction decays (FIDs) were collected for each NMR spectrum into 64K data points over a spectral width of 12,000 Hz with a relaxation delay of 6.5 μs and a mixing time of 120 ms.

### Data preprocessing and multivariate statistical analysis

The acquired ^1^H NMR spectra were phase- and baseline-corrected using the MestReNova v 9.0.1 software (Mestrelab Research S.L.). Each spectrum was referenced to TSP at δ 0.00 and peaks were aligned manually to overcome peak-shift problem [23]. With the aid of 2D NMR spectroscopy including ^1^H–^1^H correlation spectroscopy (COSY) [24], ^1^H J-Resolved and ^1^H–^13^C heteronuclear single quantum correlation (HSQC) together with previous literatures [25], the major peaks in spectra were identified and assigned to specific metabolites.

Prior to statistical analysis, these spectral data were normalized by using the probabilistic quotient normalization (PQN) method [26] to compensate for differences in overall concentrations of samples. In contrast to traditional integral normalization that takes the total integral of all signals as a function of dilution, the PQN method calculates a most probable quotient between signals of the corresponding spectrum and of a reference spectrum (normally the median spectrum of control samples) and takes it as normalization factor to account for dilution variation among samples. The spectral deconvolution method, also known as the target profiling [27], was performed to obtain the concentration of known metabolite signals in the spectra. Each spectrum was then matched in accordance with the respective signals of metabolites in standard compound library of the Chenomx NMR suite (ver 8.2, Chenomx Inc.).

Next, the quantified NMR data were exported as Microsoft Excel files and imported into the SIMCA software (version 14.1, U metrics AB, Umea, Sweden) for multivariate data analysis. The partial least squares discriminant analysis (PLS-DA) were used to examine metabolic differences due to different interventions at the molecular level. Validation of these models were performed with a seven-fold cross-validation and permutation test (200 permutations) [28]. In addition, the fold-change and Student’s *t* test with a Bonferroni correction were used for univariate analysis. Parameters from both multivariate and univariate analysis were embedded into one single volcano plot as described in previous studies [29].

### Pathway analysis

The metabolite set enrichment analysis and pathway analysis were performed using the MetaboAnalyst v4.0 (http://www.metaboanalyst.ca) [30]. In the analysis, the *Rattus norvegicus* (rat) pathway library was chosen, and the Fisher’s exact test was selected for the over-representation analysis, and relative betweenness centrality was chosen for topology analysis.

## Results

### Stool forms evaluation and plasma endotoxin assays

Microscopic examinations were conducted to study the effects of HPM or PBT treatment on morphology of intestines. As shown in Additional file 2: Figure S1, little pathological changes were observed in each group by the intestinal HE staining. The result is consistent with a previous study on intestinal permeability of IBS-D rats by administration of acetic acid [31]. The current findings indicated that the presence of diarrhea symptoms and metabolic changes are more sensitive to gastrointestinal disorder, and they occurred earlier than pathological changes in intestines.

**Fig. 1:**
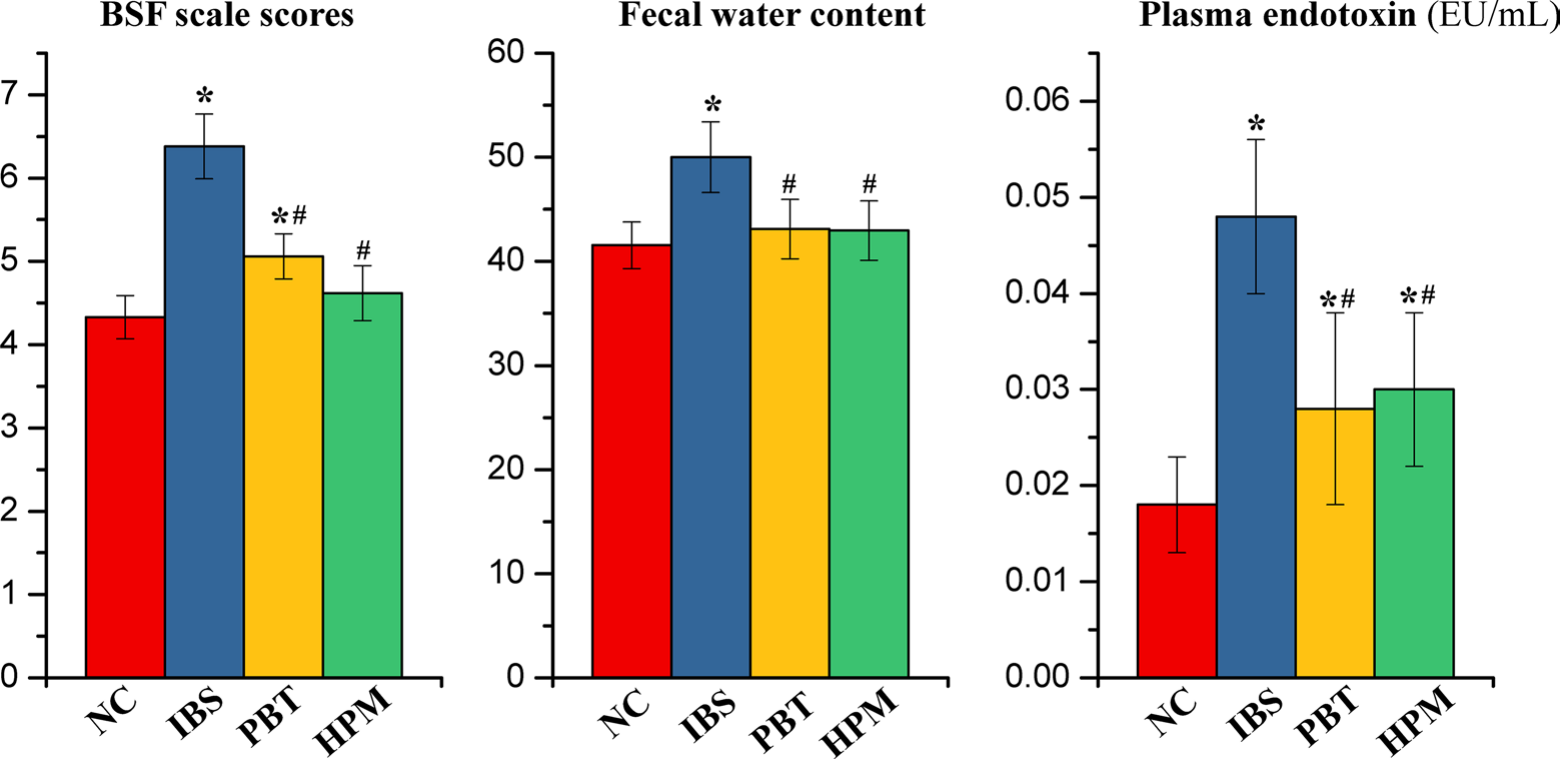
The Bristol stool forms (BSF) scale scores, fecal water content (FWC) and plasma endotoxin level for the NC, IBS, PBT and HPM groups. Error bars represent standard error of each group. *p < 0.05 statistical significant relative to the NC group; ^#^*p *< 0.05 statistical significant relative to the IBS group

Following disease modeling, the mean BSF scale scores and FWC (Fig. 1) of the IBS group were found significantly higher (*p *< 0.05) than the control NC group. This is consistent with the observation of deformed sloppy stool excreted by the IBS rats. The data indicated that the modelling of IBS-D was established successfully [32]. After treatments with either HPM or PBT, the animals showed significant decreased BSF scale scores (*p *< 0.05) and FWC (*p *< 0.05) with a firmer stool form as compared to the IBS group. By comparing the HPM and NC rats, there are no significant difference in BSF and FWC, suggesting HPM treatment is efficient in alleviating diarrhea symptoms. In contrast, PBT treatment reversed the FWC to normal but the BSF of the PBT group remained higher (*p *< 0.05) than the normal controls.

Endotoxins, also known as lipopolysaccharides (LPS), is an indicator for micro-inflammation of the intestinal mucosa that plays a critical role in the pathogenesis of IBS [33]. Plasma endotoxin levels in IBS group was found markedly increased by 166% (*p *< 0.05) after IBS modelling (Fig. 1). This finding indicated that endotoxins entered the blood circulation due to an altered permeability of the intestinal barrier in the IBS animals. Both treatments reduced the endotoxin level induced by IBS (*p *< 0.05) although the values are still higher than the NC group (*p *< 0.05). The result suggested partial reversal of intestinal inflammation due to either HPM or PBT treatment.

### ^1^H NMR profiling of feces and serum from rats

**Fig. 2:**
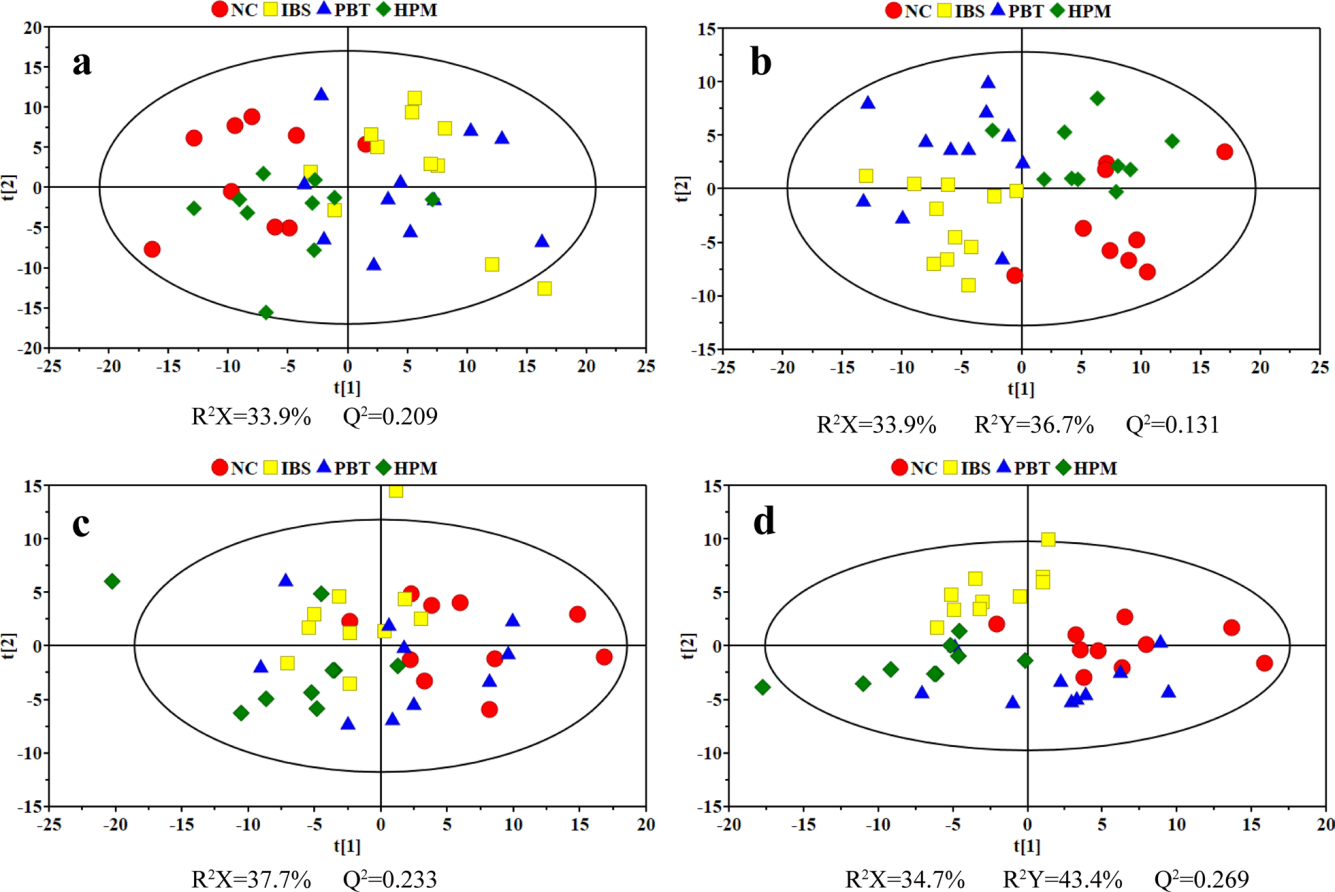
Scores plots of PCA (left column) and PLS-DA (right column) models derived from NMR datasets of feces (**a**, **b**) and serum (**c**, **d**)

Typical ^1^H NMR spectra of fecal extracts and serum are shown in Additional file 2: Figures S2 and S3. Both unsupervised (PCA) and supervised pattern recognition (PLS-DA) were applied on the NMR data of feces and serum data to observe the clustering tendency of samples due to different interventions (Fig. 2). Particularly, one fecal sample from the NC group was lost during handling and hemolysis was observed in three serum samples (one each from the IBS, HPM and PBT group), therefore they were excluded from the following analysis. For both feces and serum datasets, legible group separation with slightly overlapping can be seen in PCA score plots for the NC and IBS groups, indicating significant metabolic variations induced by colorectal administration of acetic acid and restraint stress. Points of fecal samples from rats with HPM treatment are located close to the controls whereas points from PBT groups are totally mixed with the IBS ones. Thus IBS-induced metabolic perturbations are suggested to be reversed partially towards healthy state following HPM treatment but little affected by PBT treatment. Scattered points of the PBT group in serum score plot show individual variations of metabolic patterns responding to drug intervention. However, the HPM group shows inter-group separation from the NC and IBS groups and specific intra-group gathering with one point deviating from 95% confidence interval. With combining the supervised class information (Y variable), visible separation of these four groups were improved in PLS-DA score plots (Fig. 2b, d).

### Identification of metabolic alterations in feces and serum

**Fig. 3:**
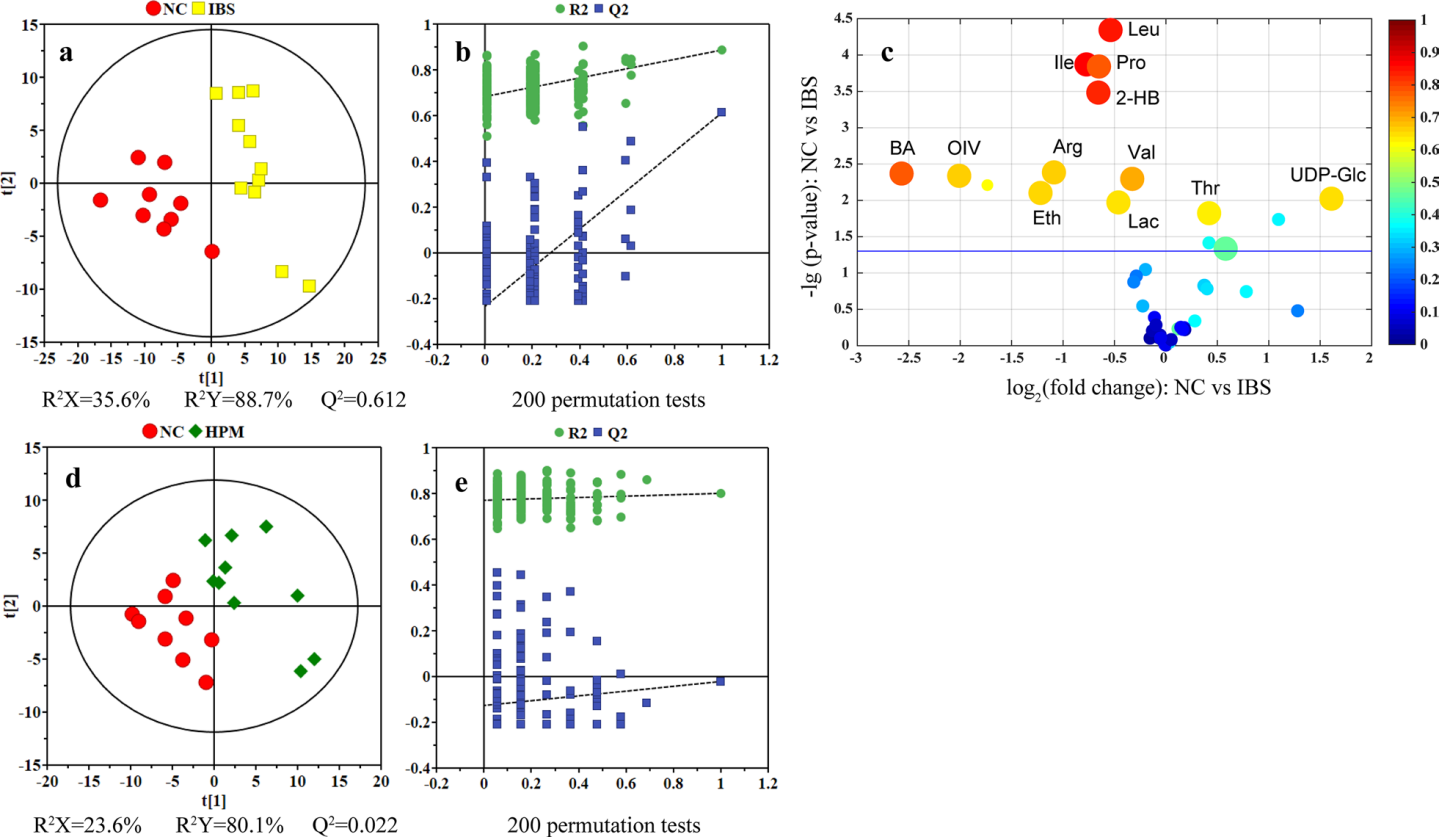
PLS-DA scores plots (left panels), their corresponding coefficient loading plots (middle panels) and volcano plots (right panels) derived from fecal NMR data. Variable importance projection (VIP) together with correlation coefficient of each model is introduced with being represented by circle size and color. VIP values are categorized into two segments: top 20% and rest 80% represented by a circle of larger or small size. Leu, leucine; Ile, isoleucine; Pro, propionate; 2-HB, 2-hydroxybutyrate; BA, bile acids; OIV, 2-oxoisovalerate; Arg, arginine; Val, valine; Eth, ethanol; Lac, lactate; Thr, threonine; UDP-Glc, uridine diphosphate glucose

**Fig. 4:**
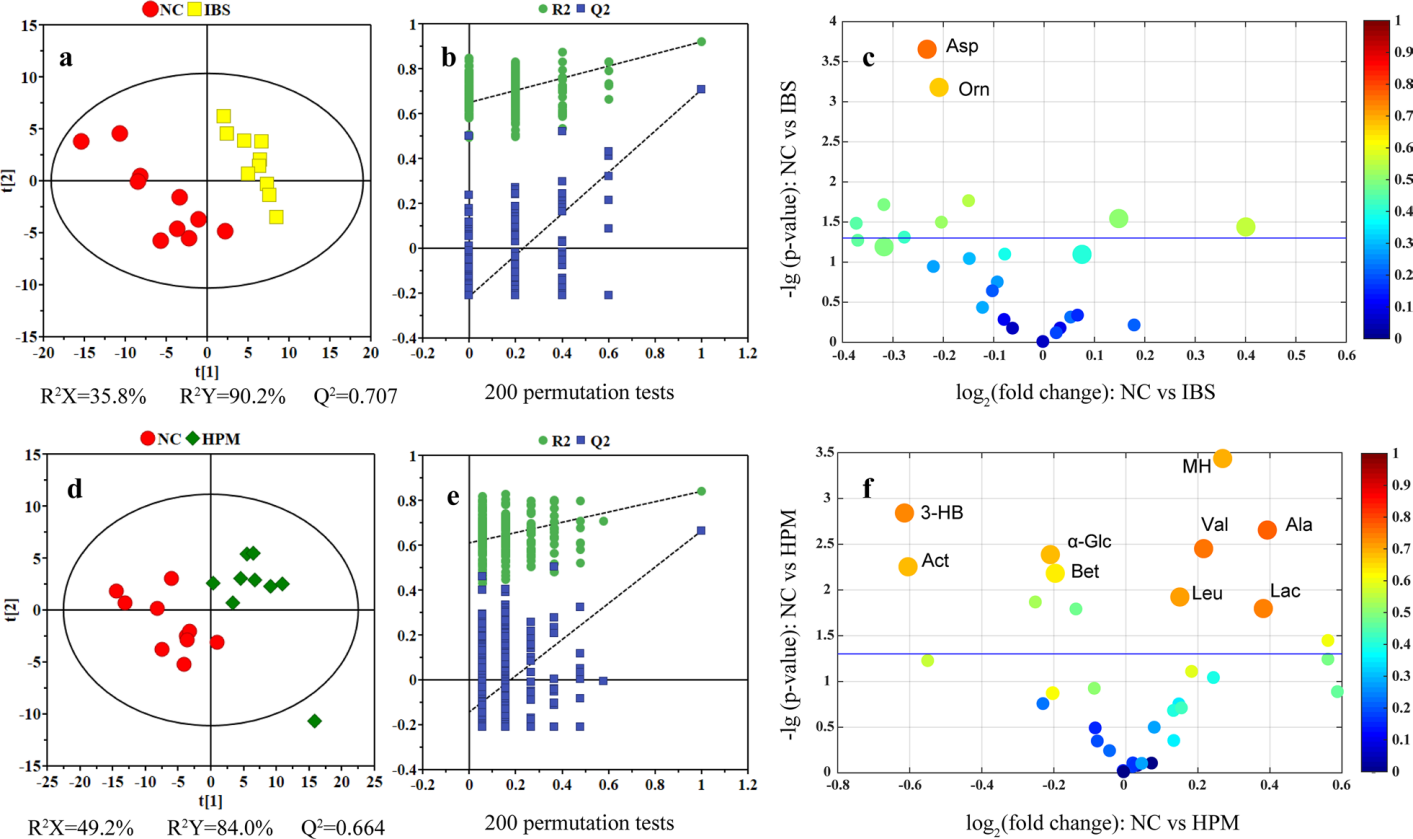
PLS-DA score plots (left panels), their corresponding coefficient loading plots (middle panels) and volcano plots (right panels) derived from serum NMR data. Leu, leucine; Val, valine; Lac, lactate; Asp, aspartate; Orn, ornithine; 3-HB, 3-hydroxybutyrate; MH, 1-methylhistidine; Act, acetone; α-Glc; α-glucose; Ala, alanine; Bet, betaine

To further investigate the metabolic impact of HPM treatment on the IBS rats, PLS-DA models were conducted on feces (Fig. 3) and serum (Fig. 4) data acquired from healthy controls, IBS rats, and IBS rats with the HPM treatment. An eightfold cross validation and random permutation tests (200 times) were carried out on the PLS-DA models to validate the robustness of the resulting PLS-DA models.

PLS-DA of fecal dataset showed a clear group separation between the NC and the IBS groups (Fig. 3a). An enhanced volcano plot, which integrates model parameters of multivariate analysis (correlation coefficient and variable importance projection) with statistical parameters of univariate (fold change and *p*-value), was used to identify candidate metabolites that contributed to the inter-group separation. Following the IBS modelling, metabolic alterations in fecal metabolome include increased concentrations of threonine and UDP-glucose together with decreased concentrations of leucine, isoleucine, proline, 2-hydroxybutyrate, valine, lactate, ethanol, arginine, 2-oxoisovalerate and bile acids (Fig. 3c).

The PLS-DA score plot of fecal samples from the NC and HPM group is shown Fig. 3d. Although separation between these two groups can be observed along the t [1] dimension, this PLS-DA model has failed in cross validation (Fig. 3e), suggesting high similarity in metabolome for both groups. For this PLS-DA model, volcano plot was not constructed as differential metabolites cannot be identified based on this unreliable discriminant model. The current results supported that HPM treatment may re-normalize the IBS-induced metabolic changes in rats.

Analysis of the blood serum dataset using PLS-DA showed good separation between the NC and the IBS groups (Fig. 4a), and between the NC and the HPM groups (Fig. 4d). These two models were found to be robust by a sevenfold cross validation and permutation test (200 times) (Fig. 4b, e). For serum metabolome, metabolic changes in the IBS group were highlighted with significant decrease of aspartate and ornithine. By contrast, the HPM group shows significant increased levels of lactate, alanine, 1-methylhistidine, valine and leucine together with decreased levels of betaine, α-glucose, 3-hydroxybutyrate and acetone.

## Discussion

**Fig. 5:**
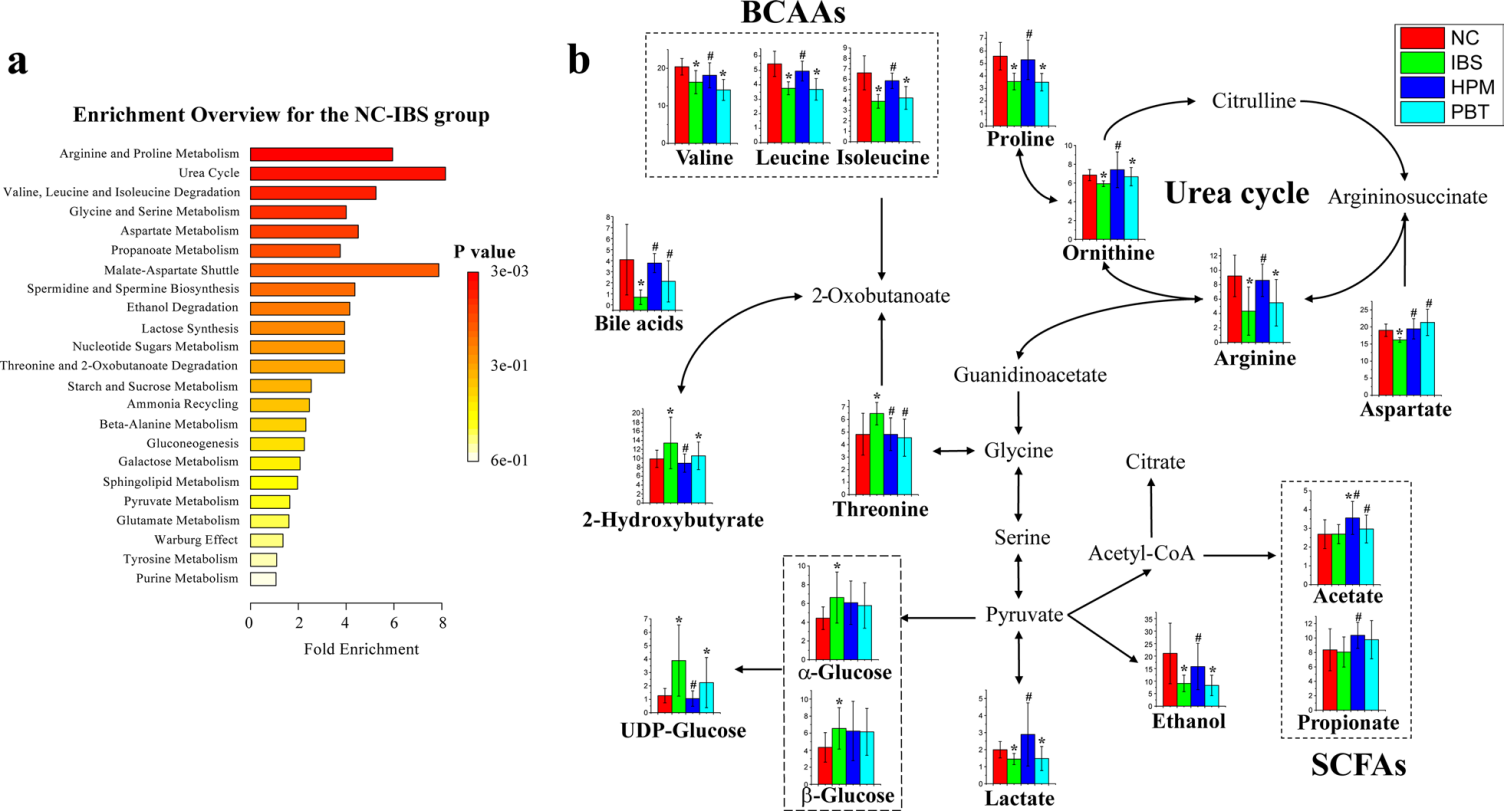
**a** Pathway enrichment analysis for the NC and IBS group; **b** summaries of metabolic pathways perturbed by IBS modeling and treatment with histograms of differential metabolites embedded into the plot. *p < 0.05 as compared to the NC group, ^#^*p *< 0.05 as compared to the IBS group

The current study used high-resolution NMR spectroscopy with multivariate statistical analysis to investigate metabolic perturbation in fecal extracts and serum samples from IBS rats with/without HPM treatment. In general, we observed relatively mild IBS-induced metabolic perturbation in the serum metabolome. In contrast, biochemical components in feces correlated directly to intestinal morphology and intestinal flora so that fecal metabolome is more sensitive to intestinal biological changes in current IBS animal model. Based on differential metabolites identified using volcano plots, an enrichment analysis of metabolic pathway was performed by the online MetaboAnalyst (Metabolomics Pathway Analysis) toolbox [30]. As shown in Fig. 5a, arginine and proline metabolism, urea cycle as well as valine, leucine and isoleucine degradation are considered to be the key three pathways changed by IBS modelling. Then, these differential metabolites are mapped onto the three metabolic pathways (Fig. 5b). Systematic metabolic alterations related to multiple biological functions were found to be changed by chemical-induced intestinal inflammation, which could be partially regulated by HPM treatment. Histograms of characteristic metabolites corresponding to the concentrations in each group were embedded into the plot to show the trends of changes.

Fecal concentration of threonine was found higher in the IBS group than the controls (*p *< 0.05). This metabolic change can be reversed by either HPM or PBT treatment (*p *< 0.05). Threonine is an essential amino acid in intestine canal that involves in the production of mucin [34] and immunoglobulin A (IgA) [35] to maintain the intestinal homeostasis. IBS-induced inflammation may facilitate the threonine utilization for synthesis of intestinal mucins and IgA [36]. Therefore, the increased threonine in feces may be due to impaired gut secretory immune system with limited luminal threonine availability upon exposure to inflammatory stimuli. This metabolic perturbation of intestinal homeostasis can be normalized by using either HPM or PBT treatment.

Aspartate, proline, ornithine and arginine are generally considered as the amino acids of arginine family. The amino acids are inter-convertible via inter-organ metabolism in mammals [37]. These four metabolites were observed to be reduced in fecal samples upon IBS modelling, and can be returned to normal after 2-week HPM treatment. Notably, aspartate is one of the major sources of ATP in mammalian enterocytes via mitochondrial oxidation [38] and play a critical role in inhibiting NODs/NF-κB and p38 signaling pathways to improve the intestinal integrity under an inflammatory condition [39]. On the other hand, arginine involves in ornithine cycle to promote urea formation, and this was found to exert vital function on intestinal inflammation via immune response and oxidative system [40]. Based on the previous reports [41], reductions in these arginine-group amino acids following IBS-modelling can be considered as an indicator of immunological stress due to intestinal injury of acetate administration.

Valine, leucine and isoleucine, known as branched-chain amino acids (BCAAs), are beneficial to maintain intestinal barrier integrity and protect intestinal mucosal permeability in absorption of nutrients [42]. Decreased concentrations of BCAAs in feces of the IBS rats may be accounted for the malabsorption of nutrients due to epithelium inflammation and injury by particular bowel pathology [43]. HPM treatment was found to significantly increase BCAAs but PBT treatment only led to minor changes compared to IBS group, supporting the holistic metabolic regulation of HPM in relieving intestinal injury and improving intestinal function.

Metabolism of bile acids has been reported to be close interacted with gut microbiome and intestinal inflammation [44]. The fecal bile acids pool composition is determined by two bacterial metabolic reactions [45]: firstly, the deconjugation of the amino acid and secondly, the transformation of primary bile acids into secondary bile acids. Dior et al. [46] reported a decreased ability for bile acids deconjugation (first step) in IBS feces compared to healthy subjects using an in vitro test. Thus, the decreased bile acids in feces of IBS rats is speculated to be correlated with the decrease in bile acid biotransformation and passively reabsorbed in the ileum. The dysregulated metabolism of bile acids was found improved by either HPM or PBT treatment.

Energy-related metabolites in the human intestine are crucial to gut microbiome and intestinal cells. Glucose metabolism in hosts can regulate T cell activation [47] and UDP-glucose can stimulate IL-8 production via the P2RY14 receptor [48]. Increased glucose and UDP-glucose levels observed in fecal samples of the IBS animals may be resulted from the disturbance in the host–microbiome system, where HPM treatment was found effective in regulating UDG-glucose level to normal.

Short-chain fatty acid (SCFAs) including acetate and propionate are readily absorbed and used as an energy source by colonocytes [49]. SCFAs provide an important defensive capacity against colorectal carcinogenesis by reducing epithelial inflammation and triggering cancer cell apoptosis via p21 activity [50]. In the current study, the concentrations of acetate and propionate in feces were comparable between the IBS group and healthy controls. However, HPM treatment led to increased levels of these two SCFAs, compared with the IBS group. This result indicated that HPM may confer protective mechanism on intestinal microbiota and host tissues upon inflammation and tumorigenesis.

## Conclusions

In conclusion, ^1^H NMR spectroscopy-based metabolomics was used to characterize the metabolome variations in blood serum and fecal samples following IBS modelling and HPM/PBT treatment. The altered metabolic pathways in IBS rats revealed the disruption of intestinal homeostasis, dysregulated immune response, malabsorption of nutrients and perturbed bile acids metabolism, which may be associated with IBS. Traditional moxibustion treatment with herbal medicine showed beneficial effects by re-normalizing IBS-induced metabolic changes.

## Additional files

**Additional file 1.** The Minimum Standards of Reporting Checklist.

**Additional file 2.** NMR spectra of fecal extracts and serum.

## Abbreviations

IBS: irritable bowel syndrome
IBS-D: diarrhea-predominant irritable bowel syndrome
IBS-C: constipation-predominant irritable bowel syndrome
IBS-M: mixed diarrhea and constipation irritable bowel syndrome
IBS-U: unspecific irritable bowel syndrome
TCM: traditional Chinese medicine
HPM: herb-partitioned moxibustion
NC: normal control
BSF: Bristol Stool Form
FWC: fecal water content
TAL: tachypleusamebocyte lysate
TSP: 3-(trimethylsilyl) propionic-2,2,3,3-d4 acid sodium salt
NMR: nuclear magnetic resonance
CPMG: Carr–Purcell–Meiboom–Gill
FID: free induction decay
COSY: correlation spectroscopy
HSQC: heteronuclear single quantum correlation
PLS-DA: partial least squares discriminant analysis
LPS: lipopolysaccharides
PCA: principal component analysis
IgA: immunoglobulin A
BCAAs: branched-chain amino acids
SCFAs: short-chain fatty acids
