# Supplementary material for: Metabolomics analysis of herb-partitioned moxibustion treatment on rats with diarrhea-predominant irritable bowel syndrome
Source: Chin Med. 2019 May 8;14:18. doi: 10.1186/s13020-019-0240-2 (PMC6505125; doi:10.1186/s13020-019-0240-2)
Supplement: Supplementary file 2 — Additional file 2. NMR spectra of fecal extracts and serum. [file 13020_2019_240_MOESM2_ESM.docx]

**Figure S1.** HE pathological section of the colon (magnification 200×)


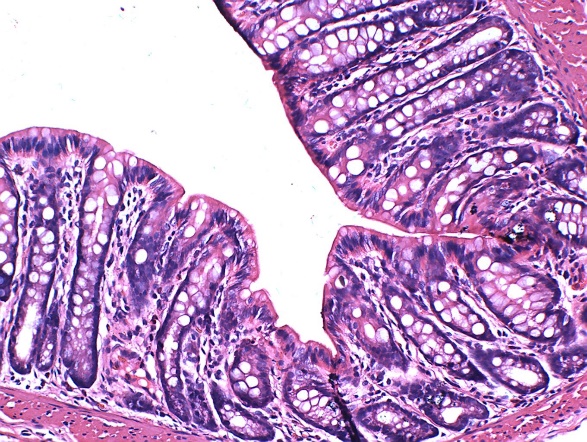

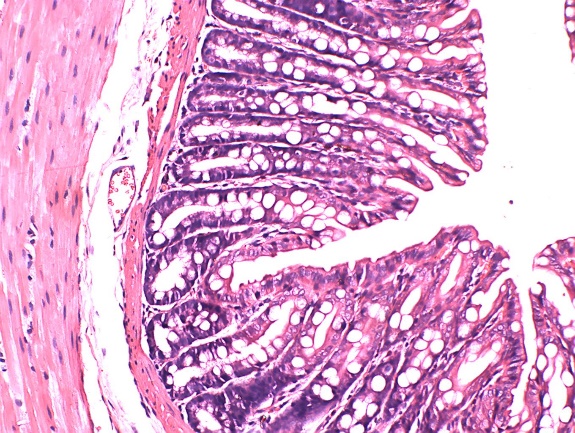


NC IBS


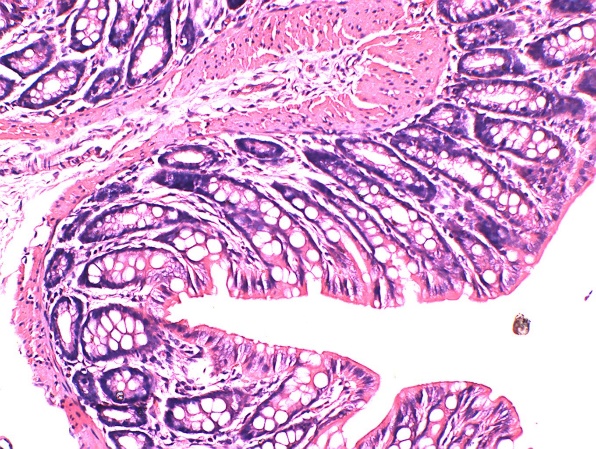

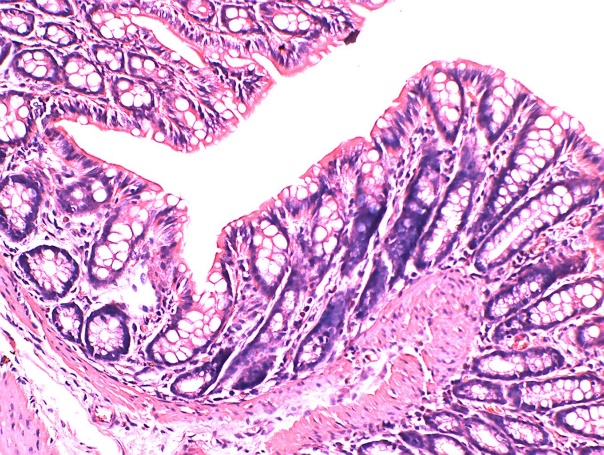


PBT HPM

**Supplemental Figure S2.** Typical ^1^H NMR spectra of fecal extracts from four groups.


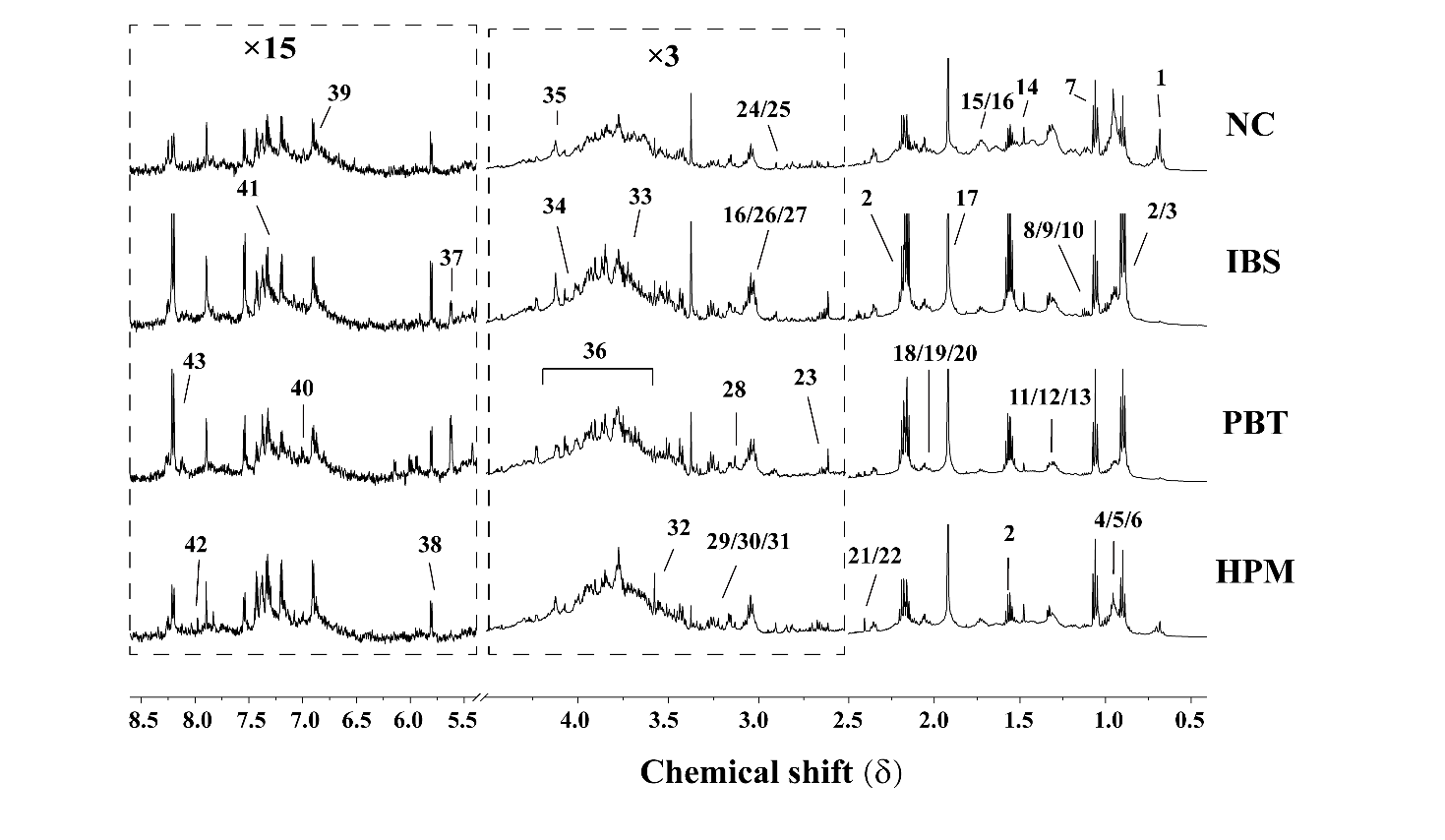


1 bile acids; 2 butyrate; 3 2-Hydroxybutyrate; 4 isoleucine; 5 leucine; 6 valine; 7 propionate; 8 isobutyrate; 9 2-Oxoisovalerate; 10 ethanol; 11 methylanine; 12 lactate; 13 threonine; 14 alanine; 15 arginine; 16 lysine; 17 acetate; 18 proline; 19 glutamate; 20 methionine; 21 succinate; 22 glutamine; 23 aspartate; 24 N,N-Dimethylglycine; 25 trimethylamine; 26 creatine; 27 creatinine; 28 malonate; 29 choline; 30 phosphocholine; 31 glycerophosphocholine; 32 glycine; 33 glycerol; 34 betaine; 35 asparagine; 36 glucose; 37 uridine diphosphate glucose; 38 uracil; 39 tyrosine; 40 para-Hydroxyphenylacetate; 41 phenylalanine; 42 urocanate; 43 hypoxanthine.

**Supplemental Figure S3.** Typical ^1^H NMR spectra of serum from four groups.


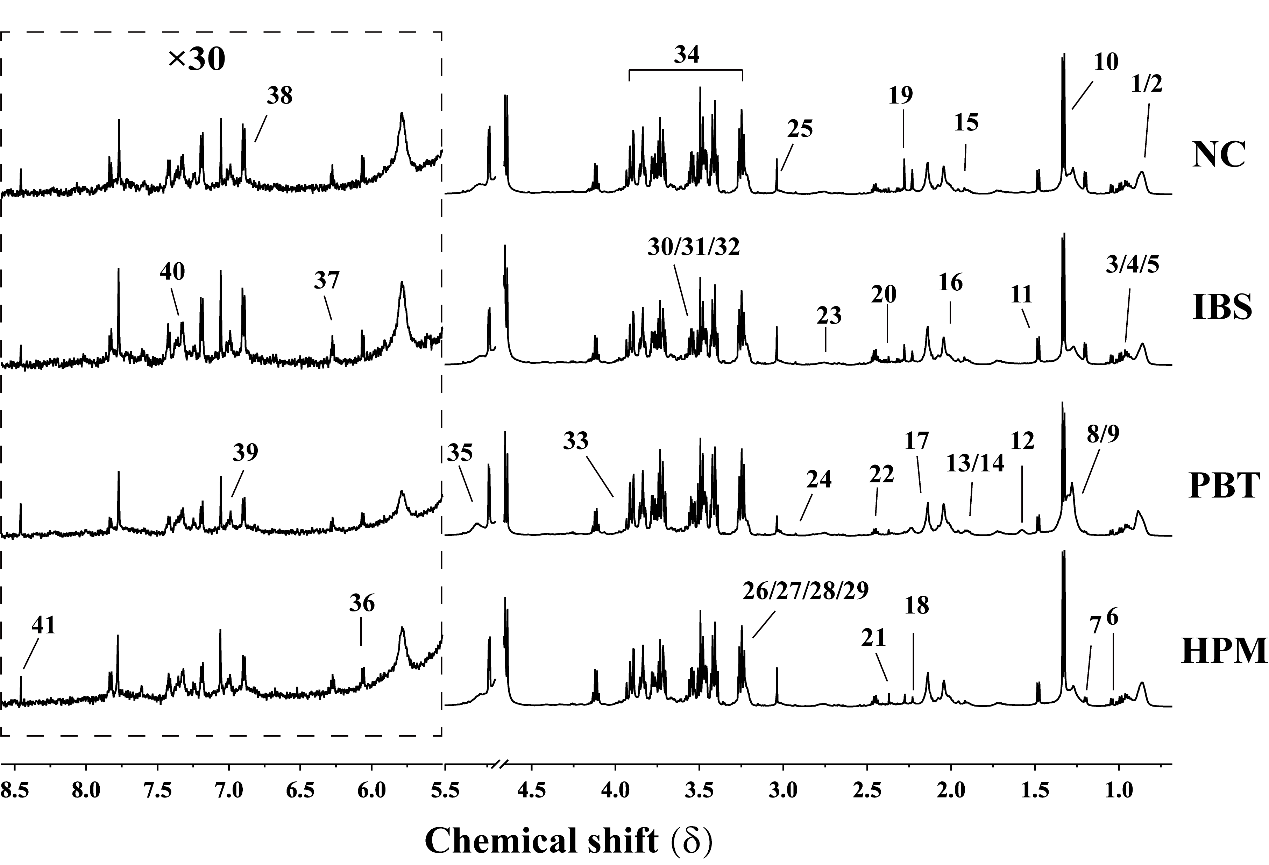


1 L1; 2 L2; 3 isoleucine; 4 leucine; 5 valine; 6 isobutyrate; 7 3-Hydroxybutyrate; 8 L3; 9 L4; 10 lactate; 11 alanine; 12 L5; 13 lysine; 14 acetate; 15 L6; 16 L7; 17 L8; 18 acetone; 19 acetoacetate; 20 glutamate; 21 pyruvate; 22 citrate; 23 L9; 24 N,N-Dimethylglycine; 25 creatine; 26 choline; 27 phosphocholine; 28 glycerophosphocholine; 29 betaine; 30 glycine; 31 threonine; 32 glycerol; 33 serine; 34 glucose; 35 L10; 36 cytidine; 37 deoxyguanosine; 38 tyrosine; 39 1-Methylhistidine; 40 phenylalanine; 42 formate.
